# Onset and Morphological Evolution of Cooperativity in Glass-Forming Liquids Composed of Anisotropically Shaped Molecules

**DOI:** 10.1021/acs.jpclett.5c01863

**Published:** 2025-07-29

**Authors:** M. Rams-Baron, A. Błażytko, M. Matussek, P. Lodowski, A. Radoń, M. Paluch

**Affiliations:** † August Chełkowski Institute of Physics, University of Silesia in Katowice, 75 Pulku Piechoty 1, 41-500 Chorzow, Poland; ‡ Institute of Chemistry, University of Silesia in Katowice, Szkolna 9, 40-006 Katowice, Poland; § Łukasiewicz Research Network, Institute of Non-Ferrous Metals, Sowińskiego 5, 44-100 Gliwice, Poland

## Abstract

Many theories attribute the dramatic slowdown in glass-forming liquid dynamics to a transition from independent to cooperative molecular motion. Although the concept of cooperative rearranging regions (CRRs) is well established, the microscopic nature of these regions and their evolution upon cooling remain subjects of active investigation. Molecules with anisotropic shapes offer a new perspective, as molecular reorientations along their long and short axes act as distinct and complementary probes of emerging cooperativity. Here, we introduce model molecules with tailored dipole orientations and demonstrate that the temperature-dependent evolution of a bimodal dielectric response captures the transition from independent to cooperative dynamics. This clear spectroscopic signature marks a significant advance in our understanding of how cooperativity emerges and evolves in glass-forming systems. Upon cooling, CRRs adopt increasingly compact morphologies, leading to a reduction in effective shape anisotropy. This transition from anisotropic to isotropic CRR geometries reveals previously unrecognized mechanisms by which anisotropy becomes suppressed during vitrification.

## Graphical abstract

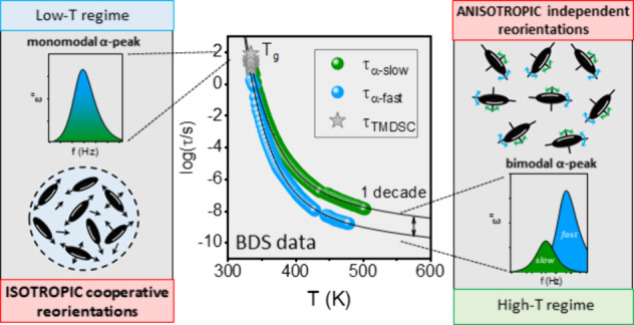

Glass-forming materials exhibit complex dynamical behavior as they approach the glass transition, with many theories linking the characteristic steep dynamical slowdown to increasing cooperativity in molecular motion.^1,2^ At high temperatures, molecular motion is typically independent, following a monoexponential relaxation and producing narrow spectral peaks in frequency-domain measurements. However, upon cooling toward the glass transition, this behavior changes fundamentally. Molecular motion becomes progressively more cooperative, leading to a distribution of relaxation times and a broadening of spectral features. Central to understanding this transition is the concept of cooperative rearrangement regions (CRRs), where groups of molecules move together in a correlated manner, contrasting with the largely uncorrelated motions at higher temperatures.^1^ While the concept of CRRs is well-established,^3,2,4,5^ the precise nature of these molecular clusters and their evolution with temperature remains a topic of ongoing investigation.^6,7^

As most studies on supercooled dynamics have focused on systems composed of spherical-like molecules, much less is known about the behavior of molecules with anisotropic shapes. As a result, a systematic understanding of how their supercooled dynamics evolve toward the glassy phase remains unclear. Unlike spherical molecules, which undergo uniform reorientation, molecules with anisotropic shapes (e.g., rod-like) exhibit distinct reorientation processes along different molecular axes, giving rise to multiple relaxation modes on different time scales. This added complexity raises important but unresolved questions: How does molecular anisotropy affect the key features of the glass transition, such as the dramatic slowdown of dynamics and the growth of cooperativity? Experimental insight into these questions has been limited. Strongly elongated molecules often form liquid crystalline phases, bypassing direct observation of the liquid-to-glass transition.^8,9^ Although simulations have offered some guidance, experimental data remain scarce.^10^ To date, no suitable model system has been available for studying how molecular shape anisotropy, along with related factors like rigidity and moments of inertia, affects reorientation dynamics in the supercooled regime.

To address this gap, we designed and synthesized two glass-forming molecules with anisotropic shapes and identical backbones but different dipole orientations, allowing us to selectively probe reorientations around the short or the long molecular axis using broadband dielectric spectroscopy (BDS). The BDS method is uniquely sensitive to dipole orientation and thus ideally suited to probe anisotropic molecular dynamics. As a general rule, BDS cannot detect a molecule’s reorientation around an axis if the dipole moment aligns parallel to that axis. By strategically modifying the placement of the polar group within the molecular backbone, we control the dipole orientation and thus which rotational degrees of freedom are accessible to BDS. The combination of dipole-sensitive spectroscopy and rational chemical design allows for unique molecular-scale resolution of anisotropic relaxation processes.

In this paper, we investigate how molecular shape anisotropy affects structural relaxation dynamics as a system approaches the glass transition. We propose an original experimental approach that offers a unique, dual-mode perspective on supercooled dynamics, where each molecular axis acts as an independent probe of reorientation. Our data reveal a clear separation of distinct relaxation modes at high temperatures, which progressively merge into a single, broadened peak upon cooling, offering direct, molecular-level evidence for the emergence of cooperative dynamics in systems composed of shape-anisotropic molecules. This transformation, from independent to collective reorientation, manifests as a distinct spectral evolution that has not been previously observed.

The chemical design of model systems designed to probe the dielectric signatures of anisotropic reorientation approaching the glassy state is shown in Figure 1b. Full synthesis and characterization details are provided in the Supporting Information (SI) file. We selected rod-like molecules in which the moment of inertia for reorientation around the short and long axes differs by a factor of 10 (see Figure 1a). For comparison, typical values for common glass formers such as propylene carbonate or toluene are approximately 3. This marked difference highlights the strong shape anisotropy in our systems, making them ideally suited to investigate anisotropic dielectric responses. As large molecules with stiff segments maintain their structure more consistently, ensuring a stable and measurable difference in inertial moments, thus, we selected systems with sufficient rigidity. The model compounds consist of two dibutylfluorene segments linked by acetylene bridges, with either a fluorophenylene (RM1) or difluorophenylene (RM4) unit introducing polarity. These rigid rod-like molecules have an aspect ratio of approximately 4.3. Although similar structures have been studied before as molecular rotors, their potential for studying the role of anisotropy in reorientation dynamics has not been exploited so far.^11,12^ In our systems, the dipole moment vector (μ) is determined by the position of fluorine substituents on the phenyl ring (Figure 1b). Density functional theory (DFT) calculations yield the following dipole moment components: for RM1, μ = 2.10 D (μ_*x*_ = 0.76 D, μ_*y*_ = −1.95 D, μ_*z*_ = 0.04 D), for RM4, μ = 3.20 D (μ_*x*_ = μ_*z*_ = 0, μ_*y*_ = −3.20 D). In RM1, the dipole moment has components along both the short and long molecular axes, providing sensitivity to the full spectrum of reorientation dynamics. In this case, the loss spectrum will reflect reorientations about both the short and long axes. In contrast, RM4 serves as a reference system in which only reorientation probed by μ oriented perpendicular to the long axis is detected. Accordingly, differences in the spectral shape of the loss peak in RM4 compared to RM1 are expected.

**Figure 1:**
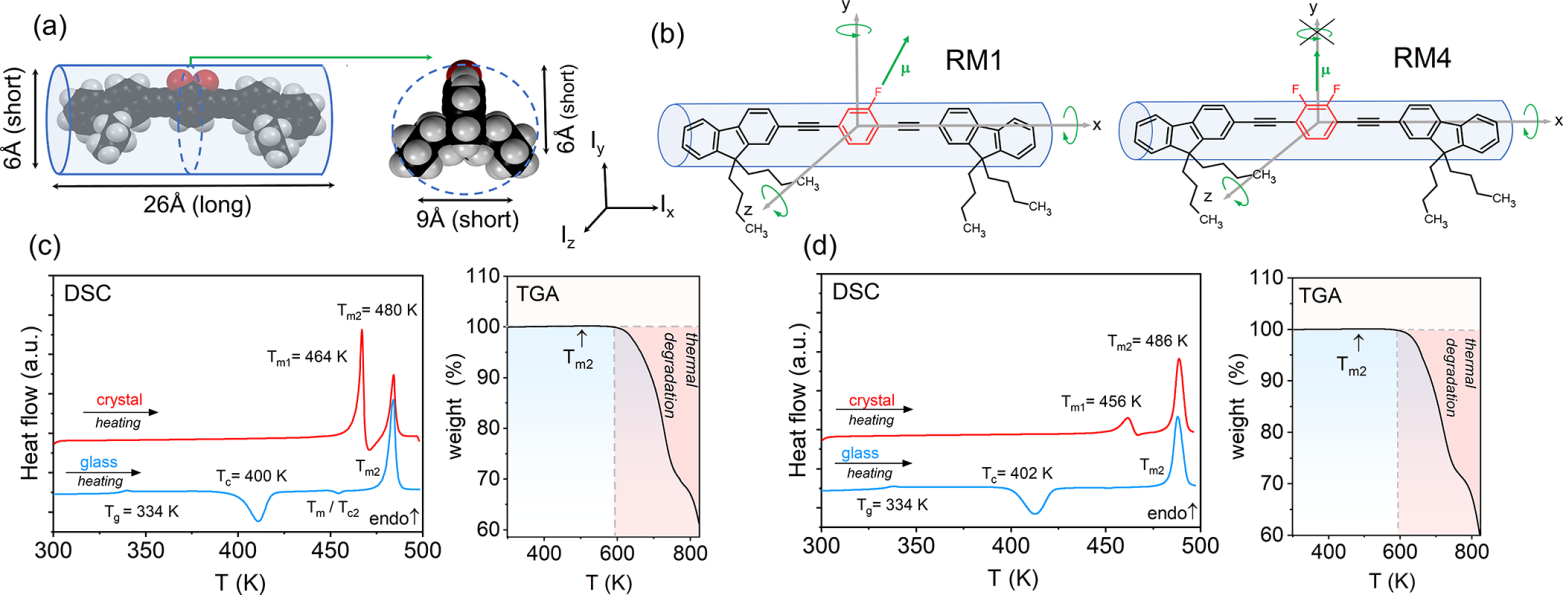
Chemical design of rod-like model molecules RM1 and RM4. (a) Space-filling model (atom labeling: C, black; H, gray; F, red) with approximated molecular length and diameter for rod-shaped structures. DFT calculated values of moment of inertia, I: for RM1, I_*x*_ = 7.90 × 10^–44^ kg·m^2^, I_*y*_ = 7.92 × 10^–43^ kg·m^2^, I_*z*_ = 7.95 × 10^–43^ kg·m^2^; for RM4, I_*x*_ = 8.55 × 10^–44^ kg·m^2^, I_*y*_ = 7.80 × 10^–43^ kg·m^2^, I_*z*_ = 7.90 × 10^–43^ kg·m^2^. (b) Line structure for RM1 and RM4. Polar regions (red) and dipole moment directions (green arrows) are shown. (c-d) DSC thermograms and TGA profiles for RM1 and RM4 from left to right, respectively. The heating rate was 10 K min^–1^. The values of glass transition temperature (*T*_g_), onset of cold crystallization (*T*_c1_ and *T*_c_), and melting temperatures (T_m1_ and T_m2_) are indicated.

To be suitable for this study, the model glass formers must supercool efficiently, with minimal crystallization and limited thermal degradation near their melting temperature (*T*_m_). Thermogravimetric analysis (TGA) and differential scanning calorimetry (DSC) were used to characterize their thermal behavior (see SI for experimental details). As shown in Figure 1c-d, both compounds form a glassy phase with a high and identical *T*_g_ = 334 K. Despite their good glass-forming ability, both show a tendency to crystallize upon heating, beginning around 400 K. For RM1, an additional event at 450 K during the second heating cycle corresponds to the melting of one form, immediately followed by recrystallization into the dominant polymorph (see Figure S1 for better resolution data). To comprehensively capture the relaxation dynamics, dielectric measurements were conducted during both cooling (from above the melting point) and heating cycles (up to the onset of crystallization). The inclusion of high-frequency dielectric data enables this approach to cover a dynamic range spanning 12 decades in time. All experimental details are provided in the SI file.

Dielectric loss spectra, ε″(f), measured above *T*_g_ for RM1 (Figure 2a) and RM4 (Figure 2b) over a range of temperatures validate our conceptual approach for designing systems in which anisotropic contributions to the dielectric response are well separated. These data show the differences in spectral shapes between RM1 and RM4. For RM1, the structural (α) relaxation peak is bimodal, with two distinct contributions labeled as slow (SP) and fast (FP) processes. Because the dipole moment in RM1 probes all aspects of molecular reorientation, we attribute these two relaxation processes to reorientations along different molecular axes. Their time scales are decoupled due to differences in the moments of inertia, as calculated via DFT (see Figure 1 caption). A larger moment of inertia leads to slower reorientation and thus longer relaxation times. Specifically, the low-frequency SP process corresponds to rotation around the short molecular axis, while the high-frequency FP component is associated with reorientation around the long axis. This interpretation is supported by Bauer’s theoretical model, which links the relaxation pre-exponential factor to the moment of inertia τ_∞_ = (2πI/*k*_B_ *T*)^0.5^, with I representing the moment of inertia and k_B_ the Boltzmann constant.^13^ This principle underlies our molecular design strategy. By increasing the size and shape-anisotropy of the studied molecules, we gained a dual perspective on the evolving dynamics, as the time scales of short- and long-axis reorientations become clearly separated. In line with the synthetic design, RM4 exhibits a monomodal ε”(f) peak. It lacks the low-frequency contribution seen in RM1, as reorientation around the short axis is dielectrically inaccessible due to dipole alignment (Figure 2b). The single peak aligns with the high-frequency component in RM1 (see Figure S2), indicating a shared molecular origin and validating our molecular-level strategy to disentangle anisotropic relaxation.

**Figure 2:**
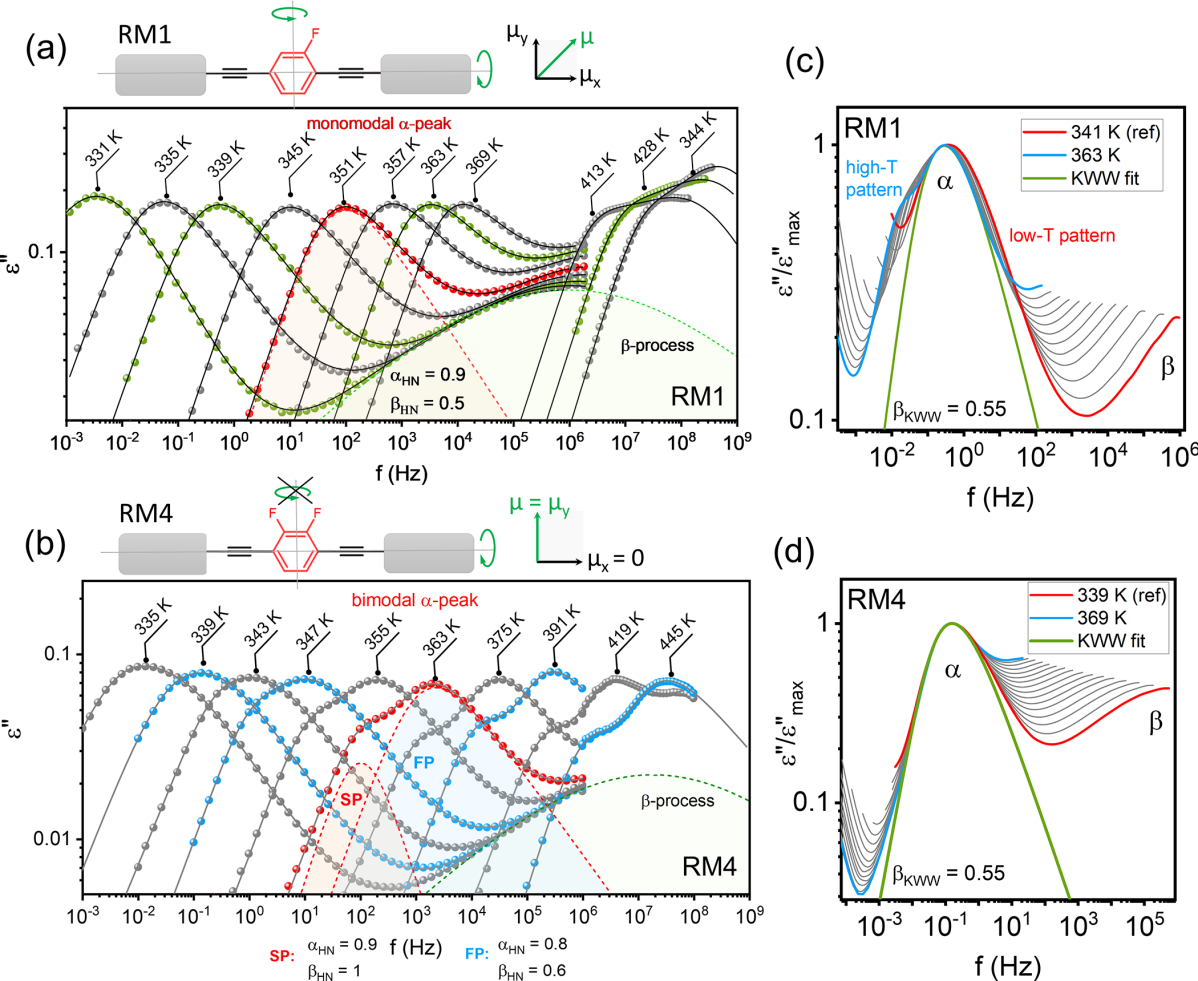
Dynamic characterization of RM1 and RM4. Dielectric loss spectra ε″(f) for RM1 (a) and RM4 (b) measured from *T*_g_ to above the melting point (dc-conductivity subtracted); symbols - experimental data, lines - fitting function. The top panel shows schematic structures with dipole vectors (green arrows) highlighting differences in molecular reorientation probed in each system. Master curves of normalized, frequency-shifted data with KWW fits for RM1 (c) and RM4 (d).

One of the most striking observations is the pronounced change in the spectral shape of RM1 loss peaks with increasing supercooling. As shown in Figure 2a, at high temperatures, the anisotropic contributions are clearly separated. However, with decreasing temperature, the spectral features gradually converge, and near *T*_g_, they merge into a broad monomodal peak. The convergence of both anisotropic modes at *T*_g_ provides clear evidence for the cooperative nature of the reorientation. As the system undergoes a transition to collective behavior, the slow and fast processes begin to merge. Such temperature evolution of the spectral shape in RM1 highlights the transition from independent to cooperative reorientation dynamics, a hallmark of supercooled dynamics. Due to the clear separation of anisotropic modes in RM1, we have a unique opportunity to observe how this complex cooperation emerges and evolves within a supercooled regime.

To analyze the ε″(f) data quantitatively, dielectric loss spectra were fitted to a superposition of the conductivity contribution and the different model functions (see the SI file for more details). Representative fit components and shape parameters are shown in Figure 2a and Figure 2b, with additional examples for RM1 provided in Figure 3a, including a high-frequency region where the α-peak overlaps with secondary relaxation processes originating from the internal rotation of the fluorophenyl ring (denoted as the β-process in Figure 2a-d). The analysis of the Havriliak–Negami shape parameters reveals that the slow relaxation mode exhibits a quasi-Debye character, with α_HN_ ≈ 1 and β_HN_ values ranging from 0.82 to 0.99. In contrast, the fast component shows a significantly broader distribution of relaxation times, characterized by β_HN_ values consistently between 0.58 and 0.64, with α_HN_ values ranging from 0.76 to 0.98. Notably, this broad distribution persists even at high temperatures. This is nicely illustrated by data collected for RM4, where only the fast process appears in the ε″(f) spectra, see Figure 2b. To further characterize the peak broadening, we scaled the data (Figure 2c-d) and used the β_KWW_ exponent from the Kohlrausch–Williams–Watts (KWW) function, φ­(t) = exp­[−(t/τ)^βKWW^].^14,15^ The widely accepted physical interpretation of β_KWW_ is that it captures dynamic heterogeneity, reflecting the distribution of relaxation times across different regions of the liquid.^16^ For RM4, β_KWW_ = 0.55 (the same behavior is observed for RM1 at high temperatures, as shown in Figure 2c), and it remains essentially temperature-independent. Typically, at high temperatures, uniform molecular motion results in a β_KWW_ exponent near unity, which gradually decreases upon cooling as the distribution of τ_α_ broadens and dynamic heterogeneity develops. Our findings challenge this idealized picture, prompting the question of how the peak shape reflects dynamic heterogeneity in this case. Another key observation is that the spectral shape varies with the orientation of the probing dipole. Reorientations probed by a dipole aligned parallel to the long molecular axis exhibit narrower relaxation time distributions than those probed by a dipole oriented perpendicularly. We recently showed that dipoles aligned parallel to the long molecular axis undergo mainly small-step diffusive motions, while dipoles oriented perpendicular to the long axis follow a more complex, two-step mechanism involving jump-like motions.^17^ However, a full explanation linking these findings to our current results remains open.

**Figure 3:**
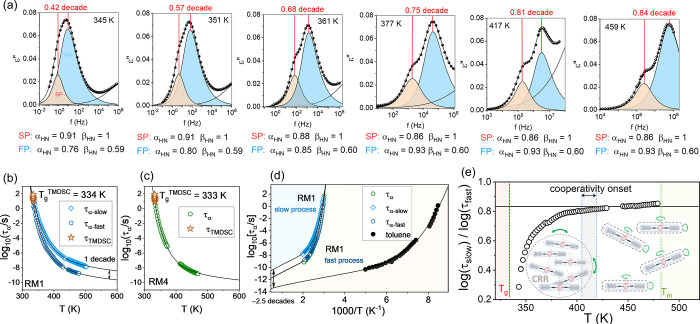
Temperature dependence of structural relaxation times in RM1 and RM4. (a) The increasing separation of anisotropic modes in RM1 with temperature indicates a loss of cooperativity. (b, c) Relaxation times for reorientations around the long and short axes in RM1 and RM4; stars indicate TMDSC values, and lines are fits to eq 1. (d) Comparison with isotropic toluene, including pre-exponential factors from eq 1. (e) The difference between logτ_α‑fast_ and log τ_α‑slow_ across different temperatures highlights the onset of cooperativity where the anisotropic mode time scales converge. The schematic representation of the evolution of the shape of reorienting regions from anisotropic geometry at high T to isotropic CRR near *T*_g_.

To identify the time scales of reorientation along the long and short molecular axes, we calculated structural relaxation times (τ_α_) using fitting parameters from the Havriliak–Negami (HN) equation. Figure 3b and Figure 3c show these relaxation times as a function of temperature for both compounds. For RM1, we refer to the relaxation times associated with reorientation around the short and long axes as τ_α‑slow_ and τ_α‑fast_, respectively. The data were fitted using a function proposed by Rössler and co-workers, which effectively parametrizes τ_α_(T) data across a wide temperature range, including both high- and low-temperature regimes.^18,19^ While previously applied to molecules with isotropic shapes, this is its first use in systems composed of anisotropically shaped molecules. This approach describes the apparent activation energy, E­(T), as the sum of a constant high-temperature term (E_∞_) and a cooperative component, E_coop_(T) ≡ E­(T)–E_∞_, which increases exponentially with cooling.^18^ The fitting uses three parameters: τ_∞_, E_∞_, and *f*, with *b* fixed at 0.10 for consistency with prior work. To improve the model’s physical relevance, we introduced a temperature-dependent pre-exponential factor, following Bauer’s suggestion.^13^ The final expression is

$$\tau =\frac{\tau_{\infty }}{\sqrt{T}}\;\exp\left[\frac{E_{\infty }}{T}+\frac{E_{\infty }}{T}\;\exp\left[-f\left(\frac{T}{E_{\infty }}-b\right)\right]\right]$$ 1

In eq 1, the fitting parameter *f* is called the generalized fragility (describing the “steepness” of E_copp_(T/T_A_), where T_A_ is a reference temperature between *T*_m_ and *T*_g_), and E_∞_ corresponds to the activation energy at high temperatures (can be calculated, e.g. by Arrhenius equation if experimental data allow this).^18,19^ The pre-exponential factor τ_∞_ is identified with the high-temperature limit of τ_α_(T), and according to Bauer’s formalism,^13^ it is interpreted as τ_∞_ = (2πI/k_B_)^0.5^. Equation 1 successfully describes the data depicted in Figure 3b-d with the following fitting parameters: for τ_α_(T) in RM4 τ_∞_ = 1.33 · 10^–11^ s/K, E_∞_ = 3565.2 K, and *f* = 90, for τ_α‑fast_(T) in RM1 τ_∞_ = 1.31 × 10^–11^ s/K, E_∞_ = 3579.0 K, and *f* = 83, and for τ_α‑slow_(T) in RM1 τ_∞_ = 2.18 × 10^–10^ s/K, E_∞_ = 3579.6 K, and *f* = 74.

As shown in Figure 3d, it is evident that τ_α_ in RM4 and τ_α‑fast_ in RM1, both probed by dipole moment oriented perpendicular to the long molecular axes, exhibit nearly identical temperature dependence, supporting the claim of a common molecular origin. The data presented in Figure 3b for RM1 confirms the merging of the time scales for short- and long-axis reorientations at *T*_g_. If the reorientation of rod-like molecules were independent across the entire supercooled regime, we would observe parallel τ_α‑fast_(T) and τ_α‑slow_(T) dependencies, as is seen at high temperatures. This would result in the observation of two distinct *T*_g_s, which is not the case in either BDS or DSC studies. As *T*_g_ is approached, the fast relaxation mode exhibits a stronger temperature dependence compared to the slow one. This is reflected in the fragility parameter, defined as *m* = [d logτ_α_/d log­(*T*_g_/T)]_T=Tg_.^20^ The less steep variation of τ_α‑slow_(T) near *T*_g_ corresponds to a lower fragility parameter (*m* = 69), compared to the higher fragility value for τ_α‑fast_(T) (m = 86), with a comparable value of *m* = 90 found for RM4. Their time scales converge at *T*_g_, as indicated by the relaxation times obtained from TMDSC measurements (stars in Figure 3b-c).

The merging of the time scales for reorientations around the long and short molecular axes provides clear evidence of a fundamental shift in the nature of molecular motion. As the system approaches the glass transition, the distinct reorientational motions (along the short and long axes) become increasingly correlated. At high temperatures, these modes are typically independent, with separate time scales. However, as the system is cooled, the number of neighboring molecules involved in reorientation increases, making relaxation progressively more cooperative. This results in a merging of the time scales for the different reorientation modes, signaling the onset of cooperative behavior. A similar behavior has been reported for another elongated molecule, itraconazole, which exhibits liquid crystalline behavior.^21^ In this case, two distinct relaxation processes are observed above *T*_g_, also associated with dipole moment reorientation around the long and short molecular axes. Upon cooling, these processes gradually merge, reflecting increasing dynamic coupling and the onset of cooperative dynamics. However, due to the presence of liquid crystalline phases, the interpretation of cooperative dynamics is more complex, as liquid crystalline ordering may influence both relaxation behavior and the morphology of dynamically correlated regions. By contrast, the model systems investigated in this study do not exhibit mesophase formation, allowing us to directly probe the intrinsic evolution of cooperative dynamics without interference from additional ordering effects.

Theoretical frameworks such as the Adam–Gibbs theory and the random first-order transition theory (RFOT) link the glass transition to the progressive decrease in configurational entropy, which in turn governs the growth of cooperative rearrangement regions (CRRs).^1,22,23^ If we assume that the decreasing difference between the relaxation times for reorientation about the short and long axis is the result of the increasing CRR, some information about this process can be extracted from our data. Our observations in RM1 suggest that as supercooling increases, the growing molecular clusters do not extend in a chain-like manner to enhance anisotropy. Instead, they preferentially form more compact, stacked assemblies, reducing shape anisotropy as cluster size increases. Near *T*_g_, isotropic shapes dominate overall morphology. If the CRRs were to build in the other direction, i.e., toward enhancing the shape anisotropy, the time scale separation between τ_α‑fast_(T) and τ_α‑slow_(T) would persist near *T*_g_. Our results, therefore, anticipate a change in the structure of CRRs, from anisotropic to isotropic as the system approaches the glass transition (see Figure 3e). To find the temperature at which the transition from independent to cooperative motion occurs, we analyze the difference between τ_α‑fast_ and τ_α‑slow_ across different temperatures. As shown in Figure 3e, the cooperativity begins to develop in the range of 405–420 K.

An additional noteworthy observation from the analysis in Figure 3d is the large difference in the high-temperature limits of the eq 1 fit. In Figure 3d we added data for toluene (taken from ref^18^), a low-molecular-weight reference, for which τ_∞_ = 7.94 × 10^–13^ s/K, E_∞_ = 1294.4 K, and *f* = 59. The comparison reveals that the high-temperature limit for τ_α_(T), τ_α‑fast_(T), and τ_α‑slow_(T) in the RM1 and RM4 corresponds to significantly longer τ_∞_. While τ_∞_ for toluene (7.94 × 10^–13^ s/K) is consistent with values reported by Rössler and co-workers for 16 other liquids,^18^ the systems examined here exhibit τ_∞_ ≥ 10^–11^ s/K due to the larger moments of inertia. The longest τ_∞_ corresponds to the reorientation around the short axis in RM1, while reorientation about the long axis results in a value shorter by an order of magnitude. These differences cannot be fully explained by simple models like the Arrhenius formalism. Only Bauer’s approach, which explicitly incorporates molecular size via the moment of inertia, provides a more accurate description of reorientation relaxation times in large rigid molecules with anisotropic shape, where the inertial effect gains more importance. The unusually long pre-exponential factors exceeding typical phonon times (∼10^–13^ s) have been observed in our previous studies on sizable glass formers.^24^ We define a molecule as sizable when τ_∞_ > 10^–12^ s (or logτ_∞_ > −12), a criterion met by the glass formers investigated here. This behavior is likely relevant for a broader class of technologically important glass-forming materials, as rigid, rod-like molecular structures are a key feature in many advanced applications. For this reason, the model molecules with anisotropic shapes examined here can be viewed as platforms for future research with broader implications for understanding dynamics in complex systems used in physics, biology, and materials science.

In conclusion, our systematic dielectric investigation of reorientation dynamics in a model glass-former composed of rod-like molecules reveals a temperature-driven evolution of bimodal loss spectra, with well-separated maxima corresponding to reorientations around the short and long molecular axes. This unprecedented spectroscopic separation of anisotropic modes offers a detailed view of how molecular dynamics transition from independent motion at high temperatures to cooperative behavior near the glass transition. In our approach, two distinct reorientation processes serve as complementary dynamic probes, each sensitive to different aspects of molecular motion. Their interplay emerges as a highly sensitive indicator of evolving dynamics, enabling us to access information beyond that provided by the analysis of a single α-relaxation process in systems with isotropic shapes. This novel perspective has opened up new possibilities for observing phenomena that were previously experimentally inaccessible, revealing, among others, that upon cooling, CRRs progressively evolve toward less anisotropic morphologies. Our findings mark a significant advancement in the field, establishing a new experimental framework for probing the fundamental mechanisms of dynamical slowdown in liquids composed of molecules with anisotropic shapes.

## Supplementary Material



## Notes

Additional experimental details, description of synthesis, material characterization data, and ^1^H and ^13^C NMR spectra for all compounds (PDF)
