# Supplementary material for: Onset and Morphological Evolution of Cooperativity in Glass-Forming Liquids Composed of Anisotropically Shaped Molecules
Source: J Phys Chem Lett. 2025 Jul 29;16(31):7953–9. doi: 10.1021/acs.jpclett.5c01863 (PMC12337139; doi:10.1021/acs.jpclett.5c01863)
Supplement: Supplementary file 1 [file jz5c01863_si_001.pdf]

Supplementary materials

Onset and Morphological Evolution of Cooperativity in Glass-Forming Liquids Composed of Anisotropically Shaped Molecules

M. Rams-Baron[1*], A. Błażytko[1], M. Matussek[2], P. Lodowski[2], A. Radoń[3], M. Paluch[1]

[1]August Chełkowski Institute of Physics, University of Silesia in Katowice, 75 Pulku Piechoty 1, 41-500 Chorzow, Poland

[2]Institute of Chemistry, University of Silesia in Katowice, Szkolna 9, 40-006 Katowice, Poland

[3]Łukasiewicz Research Network, Institute of Non-Ferrous Metals, Sowińskiego 5, 44-100 Gliwice, Poland

*corresponding author: marzena.rams-baron@us.edu.pl

1. Synthesis of anisotropic model molecules

All reagents and solvents were purchased from Merck or Tokyo Chemical Industries (TCI) and were used without further purification. Column chromatography was performed on silica gel 60 from Merck using hexane and dichloromethane. For thin layer chromatography (TLC), silica gel 60 F254 plates (Merck, Darmstadt, Germany) were used and examined under UV-light irradiation (254 and 365nm). The proton (^{1}H) and carbon (^{13}C) NMR spectra were recorded on a Bruker Avance 400 (400 MHz) or a Bruker Avance 500 (500 MHz) spectrometer. Chemical shifts (δ) are reported in parts per million (ppm) relative to traces of $CHCl_3$ (δH = 7.26 ppm, δC = 77.0 ppm) in a deuterated solvent. The following semi-products **1-4** were synthesized according to previously reported literature procedures [1,2] with minor modifications and were characterized by ^{1}H and ^{13}C NMR. All reactions were performed under an argon stream. All yields correspond to the isolated yield. The synthetic routes of intermediates and target compounds are shown in Schemes 1 and 2. In the first stage, 2-iodofluorene **1** was obtained by an iodination reaction using the I_2/H_5IO_6 system. Then, the iodinated fluorene was used in the alkylation reaction under phase transfer catalysis using tetrabutylammonium bromide as a catalyst to obtain 2-iodo-9,9-dibutylfluorene **2** in 74% yield. In the next step, the obtained iodinated and alkylated derivative **2** was subjected to Sonogashira cross-coupling reaction with trimethylsilylacetylene (TMSA) using the Pd(PPh3)4 and CuI catalytic system to obtain ethynyl derivative **3** protected with a TMS silyl group. Precursor **4** (terminal ethynyl derivative) was obtained by desilylation reaction using NaOH in MeOH and THF solution. The target compounds RM1 and RM4 were obtained *via* a Sonogashira coupling reaction between a dibrominated mono- and difluorobenzene derivatives and ethynyl intermediate **4** in the presence of $Pd(PPh_3)_4$ and CuI with good yields.

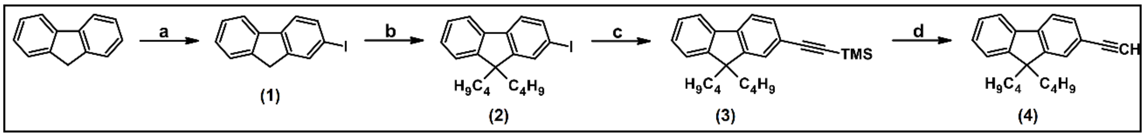

Scheme 1. Synthesis of compounds **1-4**. *Reagents and conditions:* (a) I_2, H_5IO_6, $AcOH/H_2O/H_2SO_4$, 70 °C, 6h; (b) 50% NaOH, TBAB, *n*-C_4H_9Br, DMSO, room temp., 24h; (c) TMSA (1.2 eq), $Pd(PPh_3)_4$, CuI, THF/TEA, 50 °C, 24h; (d) NaOH, MeOH/THF, room temp., 6h.

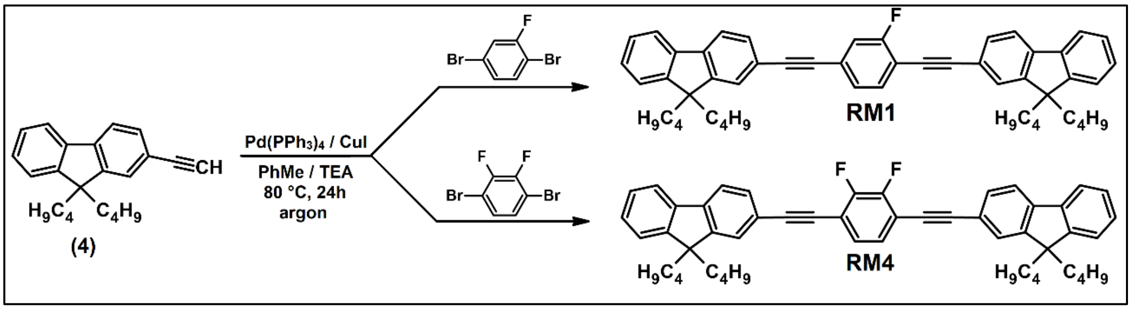

Scheme 2. The synthetic route for the preparation of compounds RM1 and RM4.

General Sonogashira cross-coupling procedure for the synthesis of RM1 and RM4

A solution of 2.5 mmol 1,4-dibromo-2-fluorobenzene (635 mg) or 1,4-dibromo-2,3-difluorobenzene (680 mg) in 90 mL degassed mixture of solvents PhMe/TEA (2:1 V/V) was stirred and bubbled with argon for 10 min. After this time, $Pd(PPh_3)_4$ (289 mg, 0.25 mmol, 10%-mol) and CuI (48 mg, 0.25 mmol, 10%-mol) were added, and the resulting mixture was bubbled with argon for another 5 min. Then, compound **4** (1.81 g, 6.0 mmol) in 15 mL TEA was injected through the septum, and the mixture was stirred at 80 °C for 24h. After the reaction was completed (monitored by TLC), the resulting mixture was filtered, and filtration was evaporated under reduced pressure. After removing the solvent, the residue was purified by column chromatography, giving the target compounds.

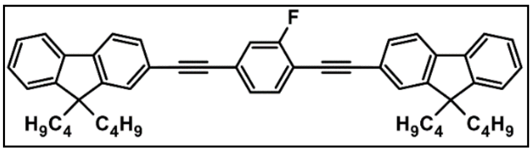

2,5-bis(9,9-dibutyl-2-ethynylfluoren)fluorobenzene (RM1). TLC: Hx/CH_2Cl_2 10:1 v/v R_f= 0.58. The crude product was purified by column chromatography (silica gel, hexane/dichloromethane 10:1 v/v). The product was obtained as a slightly yellow solid (1.51 g, 87% yield). ^{1}H NMR (500 MHz, $CDCl_3$): δ 7.76 – 7.69 (m, 4H), 7.60 – 7.52 (m, 5H), 7.40 – 7.33 (m, 8H), 2.01 (t, J = 8.3 Hz, 8H), 1.16 – 1.06 (m, 8H), 0.70 (t, J = 7.4 Hz, 12H), 0.68 – 0.55 (m, 8H). ^{13}C NMR (126 MHz, $CDCl_3$): δ 163.18, 161.18, 151.14, 151.13, 150.91, 150.87, 142.04, 142.04, 140.37, 140.33, 133.28, 133.27, 130.89, 130.83, 127.78, 127.74, 127.42, 127.39, 127.01, 126.98, 126.11, 126.10, 125.05, 124.98, 122.96, 120.90, 120.77, 120.16, 119.79, 119.75, 118.48, 118.30, 112.28, 112.15, 97.45, 97.43, 93.43, 88.22, 88.19, 82.69, 55.16,

55.14, 40.25, 25.97, 23.11, 13.88. HRMS (AP+-TOF): [M+H$^+$] calc. for $C_{52}H_{53}F$ 696.4120; found 696.4131.

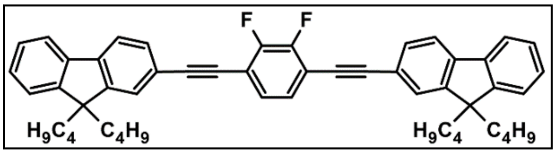

3,6-bis(9,9-dibutyl-2-ethynylfluoren)-1,2-difluorobenzene (RM4). TLC: Hx/CH_2Cl_2 10:1 v/v R_f= 0.47. The crude product was purified by column chromatography (silica gel, hexane/dichloromethane 10:1 → 7:1 v/v). The product was obtained as a pale yellow solid (1.39 g, 78% yield). ^{1}H NMR (500 MHz, $CDCl_3$): δ 7.73 – 7.66 (m, 4H), 7.57 – 7.50 (m, 4H), 7.37 – 7.31 (m, 6H), 7.29 – 7.26 (m, 2H), 1.98 (t, *J* = 8.3 Hz, 8H), 1.14 – 1.01 (m, 8H), 0.67 (t, *J* = 7.3 Hz, 12H), 0.63 – 0.51 (m, 8H). ^{13}C NMR (126 MHz, $CDCl_3$): δ 152.29, 152.15, 151.22, 150.97, 149.76, 149.61, 142.37, 140.33, 131.01, 127.88, 127.56, 127.53, 127.51, 127.04, 126.20, 123.03, 120.51, 120.25, 119.83, 114.26, 114.17, 114.11, 98.58, 98.56, 81.76, 81.73, 55.23, 40.26, 26.01, 23.14, 13.91. HRMS (AP+-TOF): [M+H$^+$] calc. for $C_{52}H_{52}F_2$ 714.4020; found 714.4037

Additional characterisation of semi-products 1-4

2-iodofluorene (**1**)

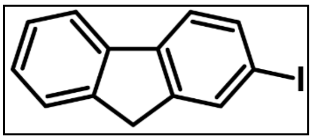

Fluorene (30 g, 180.4 mmol) was dissolved in 300 mL of the boiling mixture of acetic acid, water, and sulphuric acid (100:20:3 V/V). The mixture was cooled slowly to 70 °C, and periodic acid dihydrate (6.9 g, 30.25 mmol) and powdered iodine (15.3 g, 60.25 mmol) were added. The resulting mixture was stirred for 6h (a yellow precipitate was formed). After being cooled to room temperature, the yellow solid was collected by filtration and washed with saturated $NaHCO_3$ and water. The crude product was recrystallized from methanol (2 × 150 mL) and hexane (1 × 150 mL) to give a white solid (29.5 g, 54% yield). ^{1}H NMR (400 MHz, $CDCl_3$): δ 7.92 (s, 1H), 7.79 (d, *J* = 7.4 Hz, 1H), 7.72 (d, *J* = 8.0 Hz, 1H), 7.56 (d, *J* = 7.8 Hz, 2H), 7.42 – 7.34 (m, 2H), 3.90 (s, 2H). ^{13}C NMR (101 MHz, $CDCl_3$): δ 145.52, 142.73, 141.31, 140.80, 135.88, 134.24, 127.33, 127.01, 125.07, 121.53, 120.05, 91.85, 36.64.

2-iodo-9,9-dibutylfluorene (**2**)

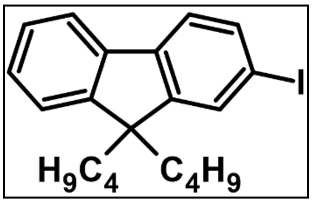

To a solution of **1** (15 g, 51.3 mmol) in DMSO (400 mL), a catalytic amount of tetrabutylammonium bromide (0.8 g, 2.6 mmol) and a 50% aqueous solution of NaOH (20 mL) were added. When the reaction mixture turned deep red, 1-bromobutane (12.2 mL, 112.8 mmol)

was added, and the resulting mixture was vigorously stirred at room temperature for 24h. After this time, the mixture was extracted with ethyl acetate. The combined organic layers were dried over anhydrous $MgSO_4$ and filtered. After removing the solvent under reduced pressure, the crude product was purified by flash column chromatography (silica gel, hexane/dichloromethane, 10:1 → 8:1 V/V) to afford **2** as a pale yellow solid (15.4 g, 74% yield). ^{1}H NMR (400 MHz, $CDCl_3$): δ 7.66 – 7.64 (m, 3H), 7.44 (d, J = 8.3 Hz, 1H), 7.34 – 7.32 (m, 3H), 2.04 – 1.83 (m, 4H), 1.18 – 1.01 (m, 4H), 0.68 (t, J = 7.3 Hz, 6H), 0.63 – 0.53 (m, 4H). ^{13}C NMR (101 MHz, $CDCl_3$): δ 153.19, 150.14, 140.82, 140.13, 135.83, 132.08, 127.69, 126.95, 122.87, 121.44, 119.83, 92.54, 55.26, 40.12, 25.92, 23.05, 13.84.

2-(trimethylsilylethynyl)-9,9-dibutylfluorene (**3**)

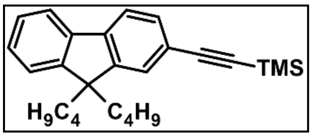

To a solution of **2** (10 g, 24.7 mmol) in 150 mL degassed THF/TEA (2:1 V/V), a catalytic mixture of $Pd(PPh_3)_4$ (1.43 g, 1.23 mmol, 5 mol-%) and CuI (235 mg, 1.23 mmol, 5 mol-%) was added. The mixture was stirred under argon for 10 min, then trimethylsilylacetylene (4.16 mL, 29.7 mmol) was injected through the septum, and the mixture was stirred at 50 °C for 24h (the progress of the reaction was monitored by silica TLC). The reaction mixture was filtered, and the filtrate was evaporated under reduced pressure. After removal of the solvent, the residue was purified by column chromatography (silica gel, hexane/dichloromethane, 10:1 V/V) to give **3** as a yellow viscous oil (6.8 g, 73% yield). ^{1}H NMR (400 MHz, $CDCl_3$): δ 7.68 – 7.62 (m, 1H), 7.60 (d, J = 7.8 Hz, 1H), 7.47 – 7.42 (m, 2H), 7.35 – 7.30 (m, 3H), 2.04 – 1.88 (m, 4H), 1.18 – 1.03 (m, 4H), 0.66 (t, J = 7.2 Hz, 6H), 0.73 – 0.58 (m, 4H), 0.32 (s, 9H). ^{13}C NMR (101 MHz, $CDCl_3$): δ 150.75, 150.63, 142.44, 141.18, 131.85, 128.23, 127.51, 126.90, 123.67, 121.94, 120.74, 120.12, 106.90, 94.45, 55.62, 40.83, 26.04, 23.67, 14.22, 0.81.

2-ethynyl-9,9-dibutylfluorene (**4**)

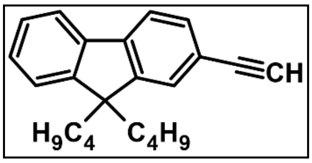

To a solution of **3** (6.0 g, 16.02 mmol) in 120 mL THF/MeOH (1:2 V/V), aqueous NaOH solution (1.2 g, 30 mmol in 6 mL water) was added. The reaction mixture was stirred at room temperature for 6h. After this time, the mixture was extracted with CH_2Cl_2. After removing the solvent under reduced pressure, the crude product was purified by flash column chromatography (silica gel, hexane) to give **4** as a dark yellow viscous oil (4.6 g, 95% yield). ^{1}H NMR (400 MHz, $CDCl_3$): δ 7.73 – 7.68 (m, 1H), 7.66 (d, J = 7.7 Hz, 1H), 7.53 – 7.46 (m, 2H), 7.38 – 7.33 (m, 3H), 3.18 (s, 1H), 2.05 – 1.91 (m, 4H), 1.18 – 1.05 (m, 4H), 0.68 (t, J = 7.2 Hz, 6H), 0.77 – 0.57 (m, 4H). ^{13}C NMR (101 MHz, $CDCl_3$): δ 151.96, 151.58, 139.98, 138.40, 131.27, 127.86, 127.16, 126.51, 122.70, 120.49, 120.39, 119.92, 84.51, 77.05, 55.52, 40.63, 26.14, 23.57, 14.29.

2. Materials and Methods

2.1 Dielectric measurements

Dielectric measurements were carried out using an Alpha impedance analyzer (Novocontrol GmbH) with a Novocool Cryosystem providing temperature control with stability of ±0.2 K to probe the complex dielectric permittivity $\varepsilon^*(\omega)$. The standard frequency range (0.1 Hz – 1 MHz) was extended down to 10^{-3} Hz to resolve slow dynamics near the glass transition. Samples were prepared by the melt-quenching method. Each compound was first heated to a melting temperature and then rapidly cooled between parallel-plate electrodes to form an amorphous sample. Samples were measured using the parallel-plate capacitor (15 mm or 10 mm diameter, depending on the frequency range) with a fixed gap of 0.05 mm maintained by quartz fibers. For RM1, measurements were performed from 335 K to 503 K in 2 K increments; for RM4, from 329 K to 503 K, also in 2 K increments. To capture high-frequency dielectric responses (1 MHz – 3 GHz), a Concept 81 dielectric spectrometer equipped with a Keysight network analyzer was used. For RM1, data were collected from 417 K to 513 K; for RM4, from 416 K to 503 K, both in 2 K steps. Dielectric loss spectra were fitted to a superposition of the conductivity contribution and the different model functions. For RM1, the bimodal $\varepsilon''(f)$ spectra were fitted using a sum of two Havriliak-Negami (HN) functions[3], while for RM4, a single HN function sufficed:

$$\varepsilon^* = \frac{\sigma}{\varepsilon_0\omega} + \varepsilon_\infty + \sum_{i=0}^{n} \frac{\Delta\varepsilon}{(1 + (i\omega\tau_{HN})^{\alpha})^{\beta}}$$

Here, $\sigma/\varepsilon_0\omega$ accounts for the dc-conductivity contribution, ε_∞ is the high-frequency permittivity limit, ω is the angular frequency, τ_{HN} is the Havriliak-Negami relaxation time, and exponents α and β are the shape parameters. To capture the high-frequency contribution from the secondary (β) process, a Cole-Cole (CC) function[4] was included in both cases. Finally, the structural relaxation time τ_α, $\tau_{\alpha\text{-slow}}$, $\tau_{\alpha\text{-fast}}$ was determined from the formula[5]:

$$\tau_\alpha = \tau_{HN} \left[sin\left(\frac{\pi\alpha}{2+2\beta}\right)\right]^{-1/\alpha} \left[sin\left(\frac{\pi\alpha}{2+2\beta}\right)\right]^{1/\alpha}$$

2.2 Calorimetric and Thermogravimetric Characterization Methods

Calorimetric measurements were conducted using a DSC 3+ differential scanning calorimeter (Mettler-Toledo), equipped with a liquid nitrogen cooling unit and an HSS8 ceramic sensor featuring a heat flux array of 120 thermocouples. Temperature and enthalpy calibrations were performed using indium and zinc standards. Approximately 5 mg of each sample was sealed in standard 40 µl aluminum crucibles. Measurements were carried out over the temperature range of 273 K to 503 K with both heating and cooling rates of 10 K/min. Crystallization (T_c) and melting (T_m) temperatures were determined from the onset of the corresponding thermal events, while the glass transition temperature (T_g) was defined at the midpoint of the step in heat flow. Temperature-modulated differential scanning calorimetry (TMDSC) was employed to determine calorimetric relaxation times in the vicinity of T_g. The TOPEM® method (Mettler-Toledo) was used, involving stochastic temperature modulation with an underlying heating rate of 0.5 K/min and a pulse amplitude of 0.5 K. The dynamic heat capacity response was analyzed over the modulation frequency range of 5 to 20 mHz. Quasi-static heat capacity values were corrected using a sapphire reference. Changes in sample mass during heating were studied using

a Mettler-Toledo TGA 2. Around 20 mg of sample was placed in 100 μl aluminum vessels and measured in a range of 303 K – 823 K with a heating rate of 10 K/min in an air atmosphere (flow rate was 50 ml/min).

2.3 Computational details

The quantum-chemical calculation were performed by applying DFT[6] level of theory with the hybrid PBE0 functional [7,8] and two basis sets, i.e. def2-SVP [9] and aug-cc-PVDZ [10]. In all calculations, the RIJCOSX approximation [11] for the Coulomb and exchange parts of the Fock matrix was used, and applied the corresponding auxiliary basis sets[12]. Calculations for the structural model were performed using the ORCA 5.0 quantum chemical package [13,14,15]. Using the def2-SVP base, the geometry of investigated molecules were fully optimized without any constraint of geometrical parameters. Based on vibrational analysis in harmonic approximation, confirms that the optimized geometries of all molecular structures corresponds to the local minima on PES. For the optimized geometries, using the aug-cc-PVDZ basis set, single-point calculations were performed to more accurately estimate dipole moment values.

3. Additional figures

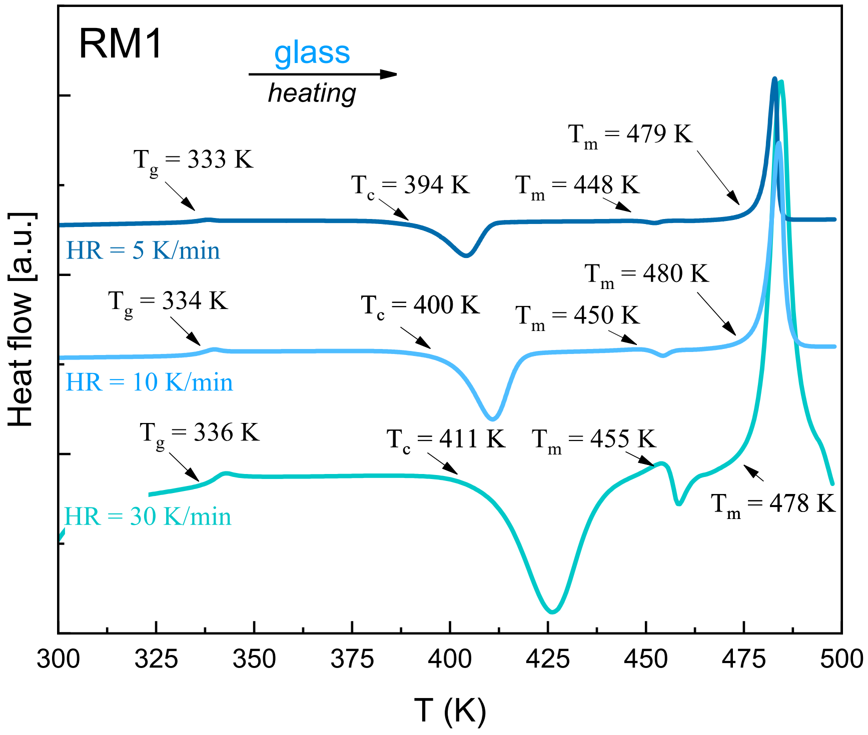

Figure S1. DSC scans for RM1 with different heating rates

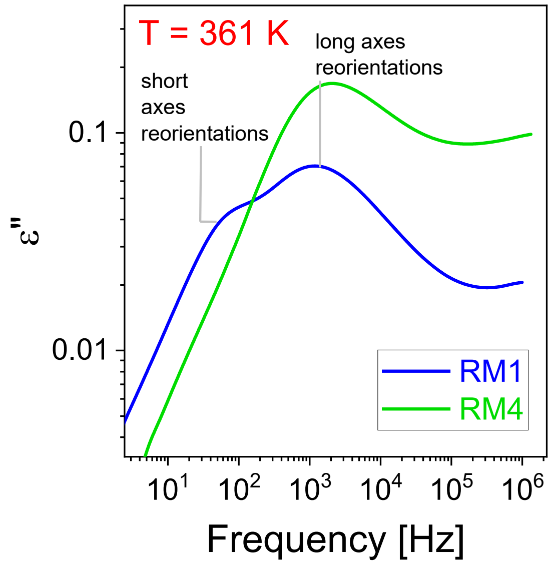

Figure S2. Comparison of the dielectric spectra for RM1 and RM4 at T = 361 K, showing that the peak of RM4 directly overlaps with the high-frequency peak of RM1 at the same temperature.

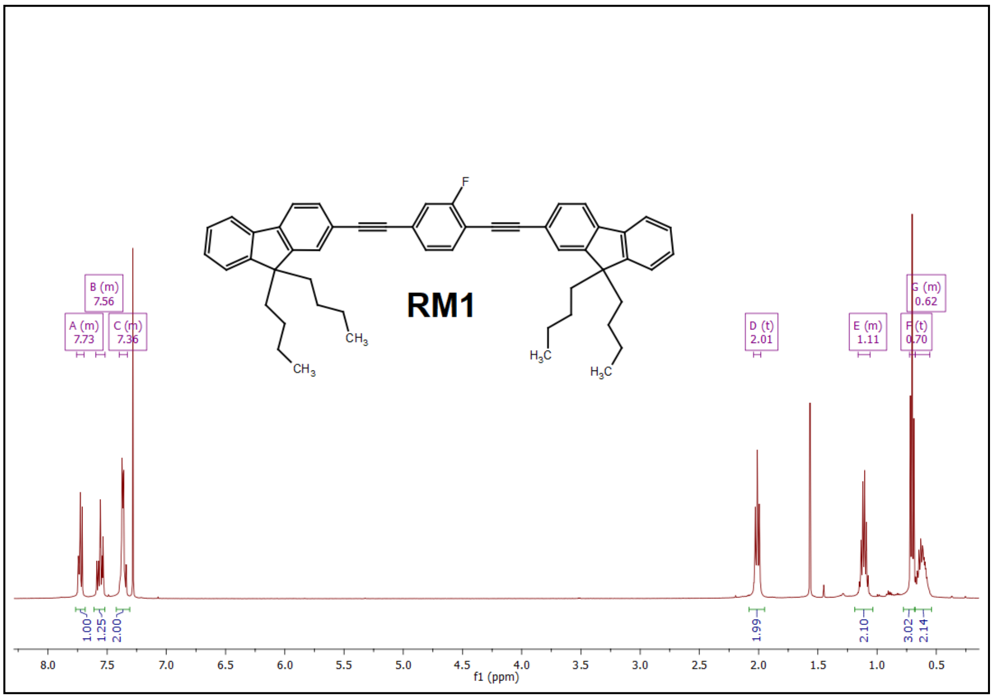

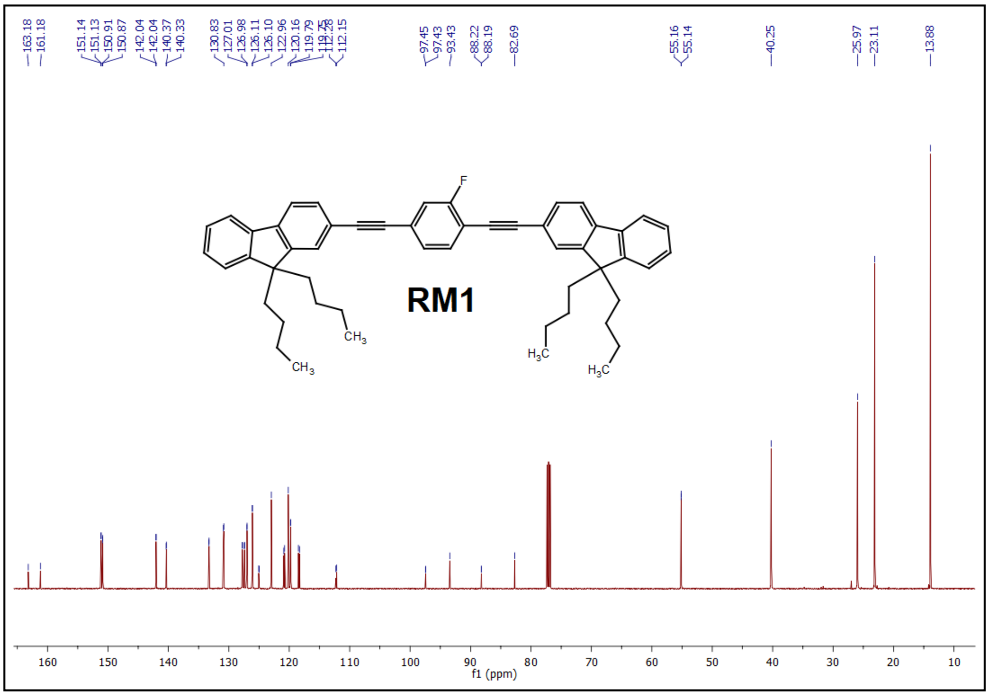
RM1
f1 (ppm)

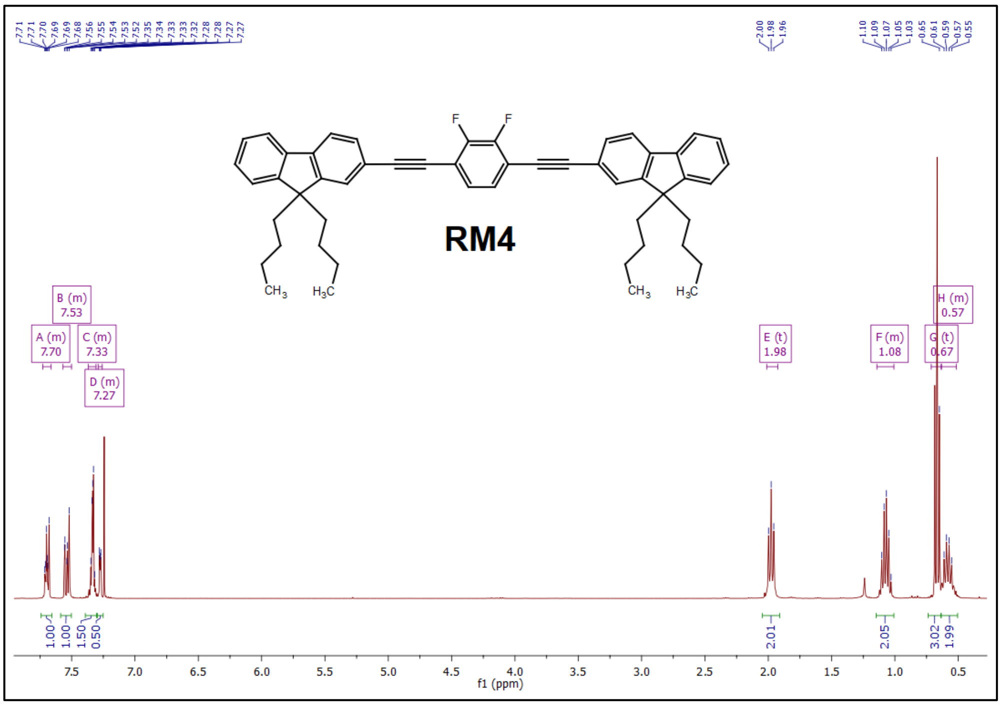
RM4
A (m) 7.70
B (m) 7.53
C (m) 7.33
D (m) 7.27
E (t) 1.98
F (m) 1.08
G (t) 0.67
H (m) 0.57
f1 (ppm)

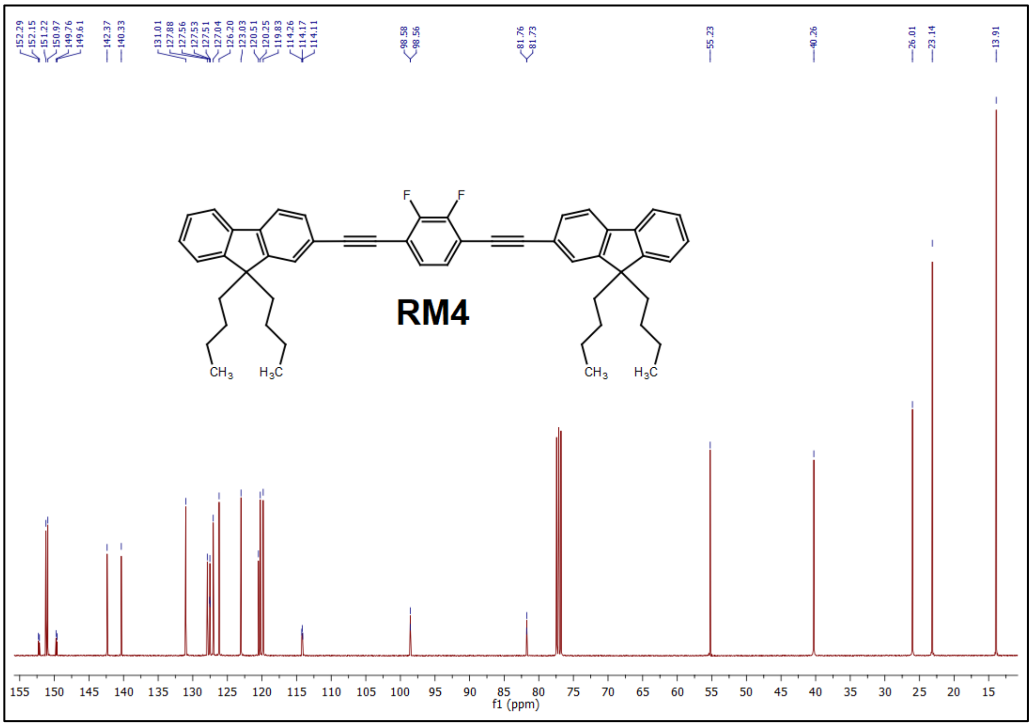

Figure S3. ^{1}H and ^{13}C NMR spectra for compounds RM1 and RM4

Mass	RA	Calc. Mass	mDa	PPM	DBE	Formula	i-FIT	i-FIT Norm	Fit Conf %	C	H	F
696.4120	100.00	696.4131	-1.1	-1.6	26.0	C52 H53 F	1031.2	n/a	n/a	52	53	1
697.4160	60.99	697.4210	-5.0	-7.2	25.5	C52 H54 F	986.2	n/a	n/a	52	54	1

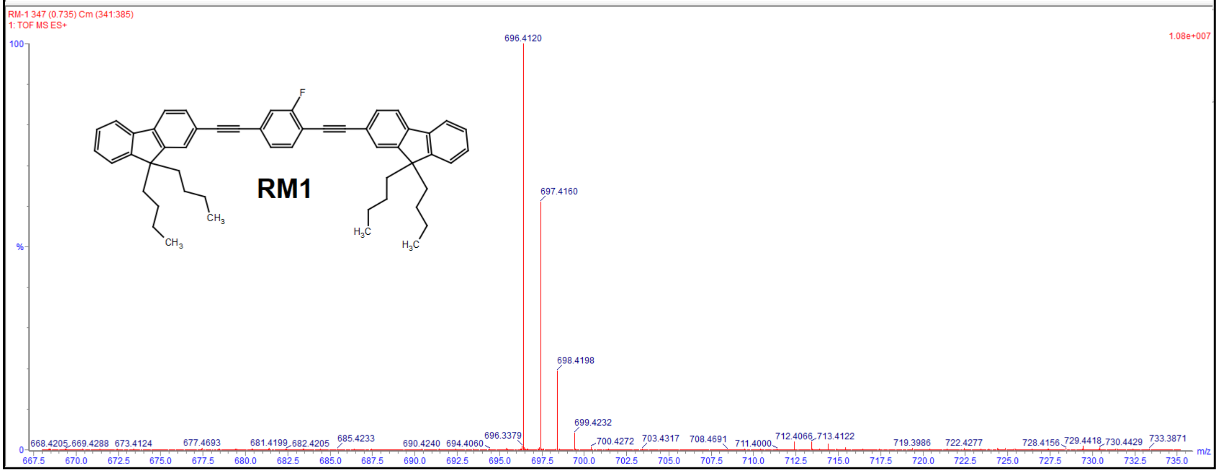

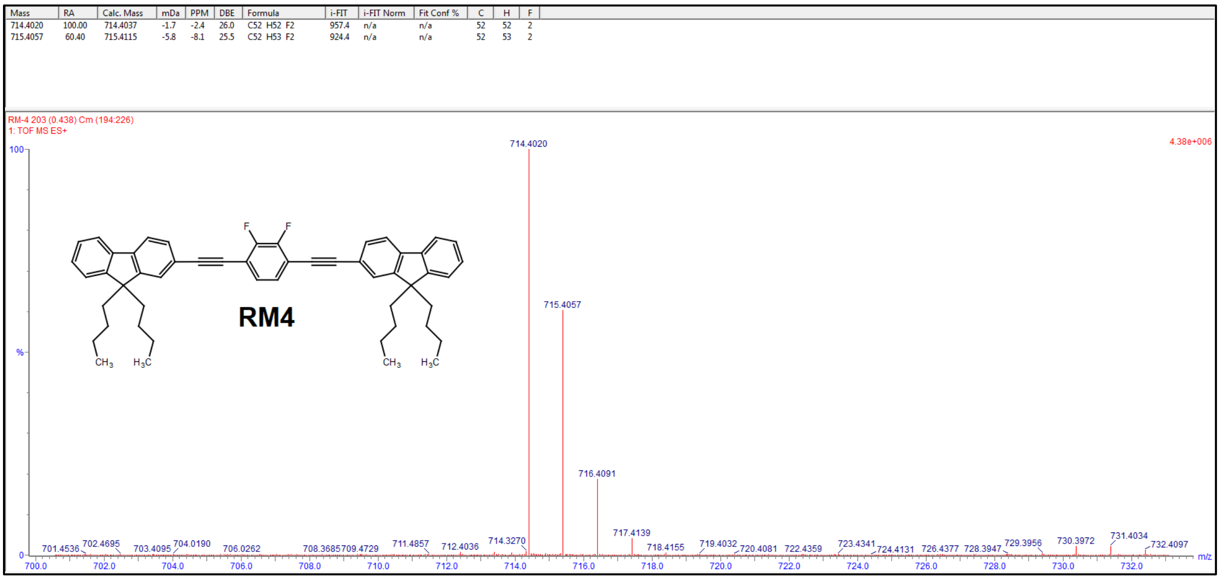

Figure S4. HRMS spectra for compounds RM1 and RM4

4. References

(1) Lee, S. H.; Nakamura, T.; Tsutsui, T. Synthesis and Characterization of Oligo(9,9-Dihexyl-2,7-Fluorene Ethynylene)s: For Application as Blue Light-Emitting Diode. *Org. Lett.* **2001**, *3* (13), 2005–2007. https://doi.org/10.1021/ol010069r.

(2) Kurpanik, A.; Matussek, M.; Szafraniec-Gorol, G.; Filapek, M.; Lodowski, P.; Marcol-Szumilas, B.; Ignasiak, W.; Małecki, J. G.; Machura, B.; Małecka, M.; Danikiewicz, W.; Pawlus, S.; Krompiec, S. APEX Strategy Represented by Diels–Alder Cycloadditions—New Opportunities for the Syntheses of Functionalised PAHs. *Chem. - A Eur. J.* **2020**, *26* (53), 12150–12157. https://doi.org/10.1002/chem.202001327.

(3) Havriliak, S.; Negami, S. A Complex Plane Representation of Dielectric and Mechanical Relaxation Processes in Some Polymers. *Polymer (Guildf).* **1967**, *8*, 161–210.

(4) Cole, K. S.; Cole, R. H. Dispersion and Absorption in Dielectrics I. Alternating Current Characteristics. *J. Chem. Phys.* **1941**, *9* (4), 341–351. https://doi.org/10.1063/1.1750906.

(5) Kremer, F.; Schonhals, A. *Broadband Dielectric Spectroscopy*; Springer-Verlag Berlin Heidelberg, 2003.

(6) Kohn, W.; Sham, L. J. Self-Consistent Equations Including Exchange and Correlation Effects. *Phys. Rev.* **1965**, *140* (4A), A1133–A1138. https://doi.org/10.1103/PhysRev.140.A1133.

(7) Adamo, C.; Barone, V. Toward Reliable Density Functional Methods without Adjustable Parameters: The PBE0 Model. *J. Chem. Phys.* **1999**, *110* (13), 6158–6170. https://doi.org/10.1063/1.478522.

(8) Perdew, J. P.; Burke, K.; Ernzerhof, M. Generalized Gradient Approximation Made Simple. *Phys. Rev. Lett.* **1996**, *77* (18), 3865–3868. https://doi.org/10.1103/PhysRevLett.77.3865.

(9) Weigend, F.; Ahlrichs, R. Balanced Basis Sets of Split Valence, Triple Zeta Valence and Quadruple Zeta Valence Quality for H to Rn: Design and Assessment of Accuracy. *Phys. Chem. Chem. Phys.* **2005**, *7* (18), 3297. https://doi.org/10.1039/b508541a.

(10) Neese, F.; Valeev, E. F. Revisiting the Atomic Natural Orbital Approach for Basis Sets: Robust Systematic Basis Sets for Explicitly Correlated and Conventional Correlated Ab Initio Methods? *J. Chem. Theory Comput.* **2011**, *7* (1), 33–43. https://doi.org/10.1021/ct100396y.
(11) Neese, F.; Wennmohs, F.; Hansen, A.; Becker, U. Efficient, Approximate and Parallel Hartree-Fock and Hybrid DFT Calculations. A "chain-of-Spheres" Algorithm for the Hartree-Fock Exchange. *Chem. Phys.* **2009**, *356* (1–3), 98–109. https://doi.org/10.1016/j.chemphys.2008.10.036.
(12) Weigend, F. Accurate Coulomb-Fitting Basis Sets for H to Rn. *Phys. Chem. Chem. Phys.* **2006**, *8* (9), 1057–1065. https://doi.org/10.1039/b515623h.
(13) Neese, F. The ORCA Program System. *Wiley Interdiscip. Rev. Comput. Mol. Sci.* **2012**, *2* (1), 73–78. https://doi.org/10.1002/wcms.81.
(14) Neese, F. Software Update: The ORCA Program System, Version 4.0. *WIREs Comput. Mol. Sci.* **2017**, *8* (1), e1327. https://doi.org/10.1002/wcms.1327.
(15) Neese, F. An Ab Initio, DFT and Semiempirical SCF-MO Package.
